# Supplementary figures and images for: Protection against experimental cryptococcosis elicited by Cationic Adjuvant Formulation 01-adjuvanted subunit vaccines
Source: PLoS Pathog. 2024 Jul 8;20(7):e1012220. doi: 10.1371/journal.ppat.1012220 (PMC11257399; doi:10.1371/journal.ppat.1012220)

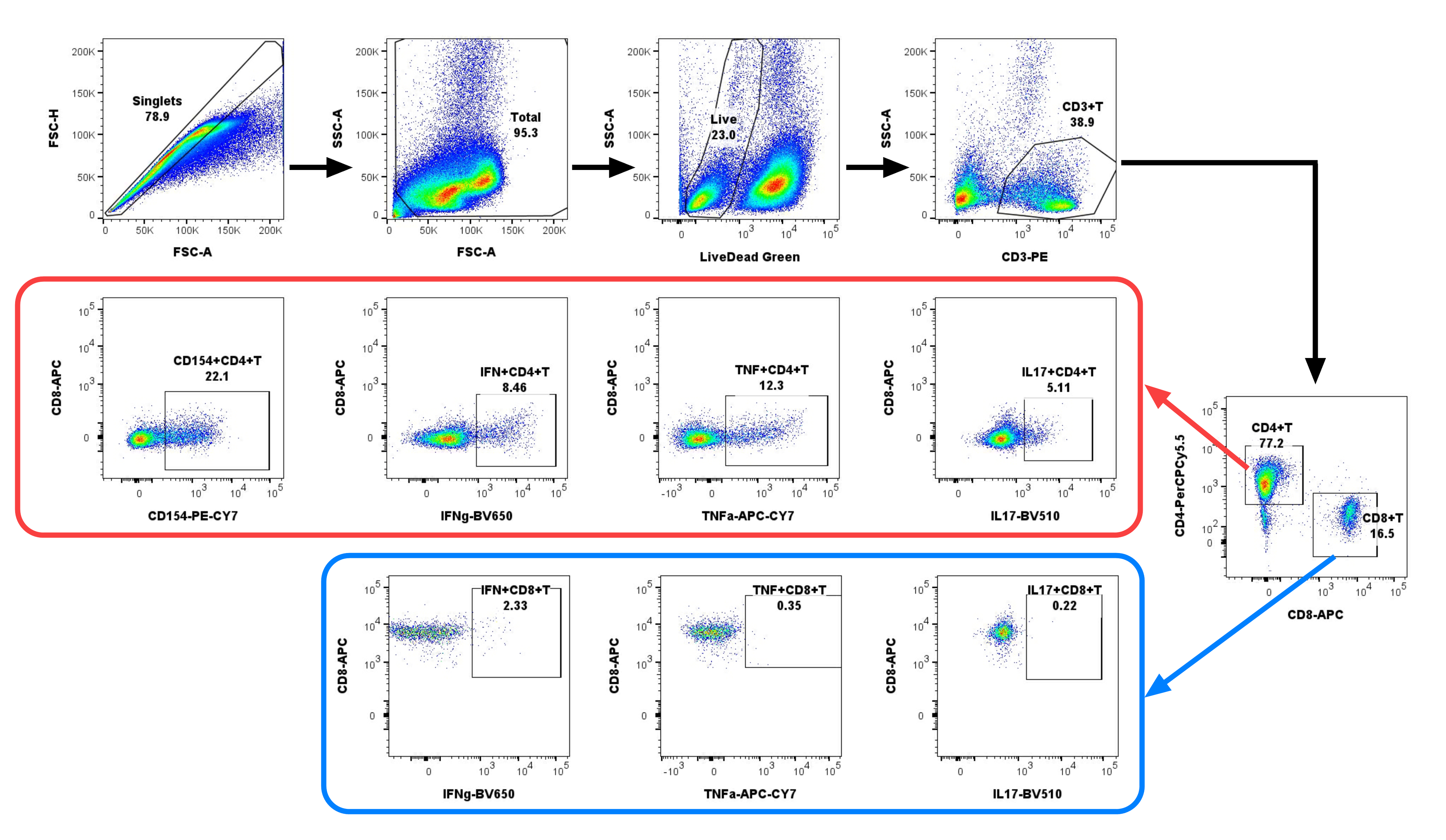

Supplement: S1 Fig — Singlet cells were gated based on forward scatter (FSC) height (FSC-H) versus area (FSC-A). Debris was excluded by considering FSC-A and side scatter area (SSC-A). Elimination of dead cells was determined through LIVE/DEAD green staining. T cells were chosen based on CD3 staining, and the CD4+CD8− subset was then selected from the CD3+ population. Subsequently, the intracellular expression of CD154, IFNγ, TNFα, and IL-17A was examined within the live CD3+CD4+CD8− gated population (highlighted in the red box). A parallel gating strategy was employed for the analysis of CD8+ T cells, with the CD4-CD8+ subset (depicted in a blue box) selected from the CD3+ population. Notably, CD154 expression was not assessed in the CD4-CD8+ population. The representative plots depict lung cells from a CAF01-Cda1+Cda2+Blp4+Cpd1Δ vaccinated mouse 10 days post-infection, stimulated ex vivo with HK C. neoformans. (TIF) [file ppat.1012220.s001.tif]

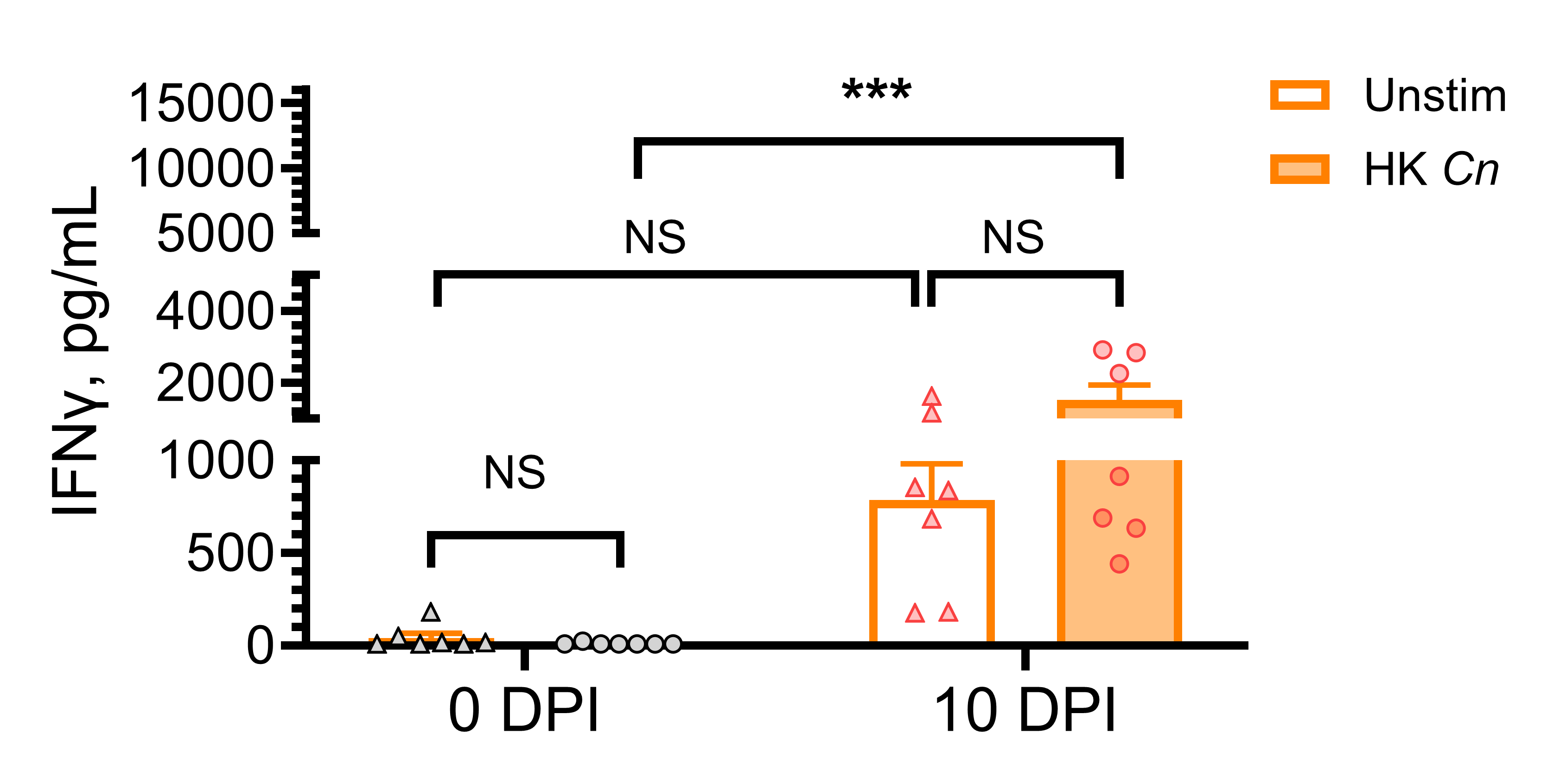

Supplement: S2 Fig — BALB/c mice were injected subcutaneously thrice at biweekly intervals with CAF01 adjuvant alone. Two weeks after the last boost, mice were orotracheally challenged with C. neoformans. Groups of mice were euthanized at 0 DPI (uninfected) and 10 DPI. Lung leukocytes were prepared as described in Methods. Cells were cultured in complete media supplemented with amphotericin B and stimulated with HK C. neoformans or left unstimulated (Unstim) for 18 h. Supernatants were collected and analyzed for IFNγ by ELISA. DPI, days post infection. HK, heat-killed. Data are presented as means ± SEM. Each dot represents the value obtained from an individual mouse. Data are from two independent experiments, each with 3–4 mice per group. Means of IFNγ levels were compared using two-way ANOVA with Šídák’s correction for multiple comparisons. NS, not statistically significant; *** P < 0.001. (TIF) [file ppat.1012220.s002.tif]
