# Supplementary material for: Protection against experimental cryptococcosis elicited by Cationic Adjuvant Formulation 01-adjuvanted subunit vaccines
Source: PLoS Pathog. 2024 Jul 8;20(7):e1012220. doi: 10.1371/journal.ppat.1012220 (PMC11257399; doi:10.1371/journal.ppat.1012220)
Supplement: S1 Table — (DOCX) [file ppat.1012220.s003.docx]

| **S1 Table. Recombinant proteins tested as vaccines** | | | | | | | |
| --- | --- | --- | --- | --- | --- | --- | --- |
| **CNAG no.**^a^ | **Name** | **Description** | **Expressed protein sequence (amino acid)** | **Vector** | **Expressed protein mass (kDa)** | ***C. gattii* ortholog** | **Human Homology^b^** |
| CNAG_05799 | Cda1 | Chitin deacetylase | 20-374 | pET19b | 41.1 | CNBG_1745 | None |
| CNAG_01230 | Cda2 | Chitin deacetylase | 20-378 | pET19b | 42.6 | CNBG_9064 | None |
| CNAG_01562 | Blp4 | Unknown | 19-321 | pET19b | 34.2 | CNBG_3874 | None |
| CNAG_00919 | Cpd1Δ^c^ | Carboxypeptidase | 22-129; 232-548 | pET19b | 51.2 | CNBG_6045 | None |
| ^a^CNAG number is from the *C. neoformans* H99 reference genome on FungiDB. Protein sequences can be found on [FungiDB](https://fungidb.org/fungidb/app). ^b^Homology to human proteins for each recombinant protein was determined using BLASTp. [Protein BLAST: search protein databases using a protein query (nih.gov).](https://blast.ncbi.nlm.nih.gov/Blast.cgi?PAGE=Proteins) ^c^Recombinant Cpd1 protein includes amino acids 22-548. The shorter version Cpd1Δ also lacks amino acids 1-21 (signal peptide) and is deleted of amino acids 130-231 so as to remove the region with homology to human cathepsin A protein ([Accession #KAI2595119.1](https://www.ncbi.nlm.nih.gov/protein/KAI2595119.1)). | | | | | | | |
