# Supplementary material for: Protection against experimental cryptococcosis elicited by Cationic Adjuvant Formulation 01-adjuvanted subunit vaccines
Source: PLoS Pathog. 2024 Jul 8;20(7):e1012220. doi: 10.1371/journal.ppat.1012220 (PMC11257399; doi:10.1371/journal.ppat.1012220)
Supplement: S2 Table — (DOCX) [file ppat.1012220.s004.docx]

| **S2 Table. Anti-mouse antibodies for intracellular staining** | | | | | |
| --- | --- | --- | --- | --- | --- |
| **Antibody** | **Company** | **Clone** | **Isotype** | **Cat #** | **Dilution** |
| CD3ε-PE | BioLegend | 145-2C11 | Armenia Hamster IgG | 100308 | 1:200 |
| CD4-PerCP/Cyanine5.5 | BioLegend | GK1.5 | Rat IgG2b, κ | 100434 | 1:200 |
| CD8a-APC | BioLegend | 53-6.7 | Rat IgG2a, κ | 100712 | 1:200 |
| CD154-PE/Cyanine7 | BioLegend | MR1 | Armenia Hamster IgG | 106512 | 1:400 |
| IFNγ-BV650 | BioLegend | XMG1.2 | Rat IgG1, κ | 505832 | 1:200 |
| TNFα-APC/Cyanine7 | BioLegend | MP6-XT22 | Rat IgG1, κ | 506344 | 1:400 |
| IL-17A-BV510 | BioLegend | TC11-18H10.1 | Rat IgG1, κ | 506933 | 1:200 |
| Rat Anti-Mouse CD16/CD32 monoclonal antibody | BD | 2.4G2 | Rat IgG2b, κ | 553142 | 1:250 |
| PE/Cyanine7 Armenia Hamster IgG Isotype Control | BioLegend | HTK888 | Armenia Hamster IgG | 400922 | 1:400 |
| BV650 Rat IgG1, κ Isotype Control | BioLegend | RTK2071 | Rat IgG1, κ | 400437 | 1:200 |
| APC/Cyanine7 Rat IgG1, κ Isotype Control | BioLegend | RTK2071 | Rat IgG1, κ | 400422 | 1:400 |
| BV510 Rat IgG1, κ Isotype Control | BioLegend | RTK2071 | Rat IgG1, κ | 400435 | 1:200 |
