# Supplementary material for: Protection against experimental cryptococcosis elicited by Cationic Adjuvant Formulation 01-adjuvanted subunit vaccines
Source: PLoS Pathog. 2024 Jul 8;20(7):e1012220. doi: 10.1371/journal.ppat.1012220 (PMC11257399; doi:10.1371/journal.ppat.1012220)
Supplement: Supplementary file 4 [file ppat.1012220.s005.docx]

S3 Table. Statistics for group comparisons in Fig 3

Comparison		Lung weight	Lung CFU	Leukocyte numbers	$CD4^+$ T cell numbers	$CD8^+$ T cell numbers
		P	***P***	***P***	***P***	***P***
0 DPI	Unvac VS 2Ag	>0.9999	-	>0.9999	>0.9999	>0.9999
0 DPI	Unvac VS 4Ag	>0.9999	-	>0.9999	>0.9999	>0.9999
0 DPI	2Ag VS 4Ag	>0.9999	-	>0.9999	>0.9999	>0.9999
10 DPI	Unvac VS 2Ag	0.4351	0.0012	<0.0001	<0.0001	<0.0001
10 DPI	Unvac VS 4Ag	0.0139	0.0002	<0.0001	<0.0001	<0.0001
10 DPI	2Ag VS 4Ag	0.9664	0.9551	0.1110	0.1232	0.9101
70 DPI	2Ag VS 4Ag	>0.9999	0.2091	>0.9999	>0.9999	0.9993
0 DPI VS 10 DPI	2Ag	<0.0001	-	<0.0001	<0.0001	<0.0001
0 DPI VS 70 DPI	2Ag	0.0628	-	>0.9999	>0.9999	>0.9999
10 DPI VS 70 DPI	2Ag	0.1312	0.0227	<0.0001	<0.0001	<0.0001
0 DPI VS 10 DPI	4Ag	<0.0001	-	<0.0001	<0.0001	<0.0001
0 DPI VS 70 DPI	4Ag	0.1117	-	>0.9999	>0.9999	>0.9999
10 DPI VS 70 DPI	4Ag	0.0003	0.0084	<0.0001	<0.0001	<0.0001
0 DPI VS 10 DPI	Unvac	0.0052	-	0.8128	0.9970	0.9996

Means of lung weights were compared using one-way ANOVA with Šídák correction for multiple comparisons. Median lung CFUs within groups were compared using the Mann Whitney U test. Leukocyte, $CD4^+$ T cell, and $CD8^+$ T cell numbers were also compared using one-way ANOVA with Šídák correction for multiple comparisons. -, not applicable. *P* < 0.05 was considered significantly different and marked with red color.
