# Supplementary material for: Protection against experimental cryptococcosis elicited by Cationic Adjuvant Formulation 01-adjuvanted subunit vaccines
Source: PLoS Pathog. 2024 Jul 8;20(7):e1012220. doi: 10.1371/journal.ppat.1012220 (PMC11257399; doi:10.1371/journal.ppat.1012220)
Supplement: Supplementary file 5 [file ppat.1012220.s006.docx]

S4 Table. Statistics for group comparisons of cytokine-producing CD4$^+$ T cells in Fig 4

Comparison		0 DPI				10 DPI				70 DPI			
		CD154$^+$CD4$^+$ T	IFNγ$^+$CD4$^+$ T	TNFα$^+$CD4$^+$ T	IL-17$^+$CD4$^+$ T	CD154$^+$CD4$^+$ T	IFNγ$^+$CD4$^+$ T	TNFα$^+$CD4$^+$ T	IL-17$^+$CD4$^+$ T	CD154$^+$CD4$^+$ T	IFNγ$^+$CD4$^+$ T	TNFα$^+$CD4$^+$ T	IL-17$^+$CD4$^+$ T
		P	***P***	***P***	***P***	***P***	***P***	***P***	***P***	***P***	***P***	***P***	***P***
Unstim	Unvac VS 2Ag	0.0166	0.4438	0.3947	0.9796	0.0001	0.1499	0.2497	0.5017	-	-	-	-
Unstim	Unvac VS 4Ag	0.0173	0.1688	0.3650	0.8092	<0.0001	0.0618	0.0411	0.3510	-	-	-	-
Unstim	2Ag VS 4Ag	0.9998	0.7854	0.9980	0.8892	0.0101	0.9337	0.6957	0.9732	0.9228	0.9538	0.9948	0.5700
HK Cn	Unvac VS 2Ag	0.3086	0.2869	0.6624	0.9998	<0.0001	<0.0001	<0.0001	0.0009	-	-	-	-
HK Cn	Unvac VS 4Ag	0.0482	0.0185	0.2070	0.9904	<0.0001	<0.0001	<0.0001	<0.0001	-	-	-	-
HK Cn	2Ag VS 4Ag	0.6030	0.3947	0.6685	0.9929	0.0947	0.2167	0.0283	0.7160	0.5115	0.1758	0.3543	0.2128
Unvac	Unstim VS HK Cn	0.9976	0.9969	0.9125	0.8959	>0.9999	>0.9999	>0.9999	0.9999	-	-	-	-
2Ag	Unstim VS HK Cn	0.2111	0.9696	0.9996	0.9572	0.1633	0.0015	0.0004	0.0321	0.7872	0.7641	0.7982	0.9639
4Ag	Unstim VS HK Cn	0.7905	0.6527	0.7611	0.9992	0.6174	<0.0001	<0.0001	0.0041	0.2745	0.0486	0.0922	0.6113

Means of cytokine-producing CD4$^+$ T cells were compared using two-way ANOVA with Tukey or Šídák correction for multiple comparisons. Unvac, unvaccinated. DPI, days post infection. HK, heat-killed. 2-Ag, vaccination with CAF01-Cda1+Cda2. 4-Ag, vaccination with CAF01-Cda1+Cda2+Blp4+Cpd1Δ. -, not applicable. P < 0.05 was considered significantly different and highlighted with red color.
