# Supplementary material for: Protection against experimental cryptococcosis elicited by Cationic Adjuvant Formulation 01-adjuvanted subunit vaccines
Source: PLoS Pathog. 2024 Jul 8;20(7):e1012220. doi: 10.1371/journal.ppat.1012220 (PMC11257399; doi:10.1371/journal.ppat.1012220)
Supplement: Supplementary file 6 [file ppat.1012220.s007.docx]

S5 Table. Statistics for group comparisons of cytokine-producing $CD8^+$ T cells in Fig 6

Comparison		0 DPI			10 DPI			70 DPI		
		$IFN\gamma^+CD8^+$ T	$TNF\alpha^+CD8^+$ T	$IL\text{-}17^+CD8^+$ T	$IFN\gamma^+CD8^+$ T	$TNF\alpha^+CD8^+$ T	$IL\text{-}17^+CD8^+$ T	$IFN\gamma^+CD8^+$ T	$TNF\alpha^+CD8^+$ T	$IL\text{-}17^+CD8^+$ T
		P	***P***	***P***	***P***	***P***	***P***	***P***	***P***	***P***
Unstim	Unvac VS 2Ag	0.9699	0.7482	0.9889	0.3888	0.0169	0.1127	-	-	-
Unstim	Unvac VS 4Ag	0.9751	0.6416	0.8105	0.0951	0.0006	0.1851	-	-	-
Unstim	2Ag VS 4Ag	0.9997	0.9806	0.6914	0.7428	0.5339	0.9445	0.9898	0.9741	0.9267
HK Cn	Unvac VS 2Ag	0.9683	0.9993	0.9714	0.0177	0.0075	0.0197	-	-	-
HK Cn	Unvac VS 4Ag	0.9887	0.9883	0.8137	<0.0001	<0.0001	0.0671	-	-	-
HK Cn	2Ag VS 4Ag	0.9947	0.9931	0.6789	0.0003	0.0178	0.8205	0.4922	0.2837	0.6058
Unvac	Unstim VS HK Cn	0.9990	>0.9999	0.9672	0.9951	0.9406	0.9995	-	-	-
2Ag	Unstim VS HK Cn	0.9786	0.8588	0.9758	0.2671	0.8234	0.8871	0.6811	0.9267	0.8085
4Ag	Unstim VS HK Cn	0.9936	0.8184	0.9719	<0.0001	0.0311	0.9683	0.0924	0.6058	0.9367

Means of cytokine-producing $CD8^+$ T cells were compared using two-way ANOVA with Tukey or Šídák correction for multiple comparisons. Unvac, unvaccinated. DPI, days post infection. HK, heat-killed. 2-Ag, vaccination with CAF01-Cda1+Cda2. 4-Ag, vaccination with CAF01-Cda1+Cda2+Blp4+Cpd1Δ. -, not applicable. $P < 0.05$ was considered significantly different and highlighted with red color.
