# Supplementary material for: Protection against experimental cryptococcosis elicited by Cationic Adjuvant Formulation 01-adjuvanted subunit vaccines
Source: PLoS Pathog. 2024 Jul 8;20(7):e1012220. doi: 10.1371/journal.ppat.1012220 (PMC11257399; doi:10.1371/journal.ppat.1012220)
Supplement: Supplementary file 7 [file ppat.1012220.s008.docx]

S6 Table. Statistics for group comparisons of IFNγ levels in Fig 7

Comparison		0 DPI			10 DPI			70 DPI		
		**Lung**	**Spleen**	**PBMC**	**Lung**	**Spleen**	**PBMC**	**Lung**	**Spleen**	**PBMC**
		P	***P***	***P***	***P***	***P***	***P***	***P***	***P***	***P***
Unstim	Unvac VS 2Ag	0.2485	0.8285	0.9257	0.8202	>0.9999	>0.9999	-	-	-
Unstim	Unvac VS 4Ag	0.2854	0.9945	0.9923	0.8609	0.9974	0.9990	-	-	-
Unstim	2Ag VS 4Ag	0.9953	0.8785	0.8752	0.9948	0.9977	0.9984	0.9673	0.9950	0.0269
HK Cn	Unvac VS 2Ag	0.6651	0.0206	0.3440	0.0003	0.0369	0.3067	-	-	-
HK Cn	Unvac VS 4Ag	0.0962	0.0081	0.9844	<0.0001	0.0004	0.0535	-	-	-
HK Cn	2Ag VS 4Ag	0.4222	0.9318	0.2671	0.0362	0.2357	0.6081	0.2944	0.8503	0.0013
Unvac	Unstim VS HK Cn	0.1143	>0.9999	0.9977	>0.9999	0.9772	0.9949	-	-	-
2Ag	Unstim VS HK Cn	0.9846	0.0953	0.4406	0.0021	0.0163	0.2100	0.5742	0.0269	>0.9999
4Ag	Unstim VS HK Cn	0.3713	0.0118	0.9997	<0.0001	<0.0001	0.0204	0.0321	0.0013	0.4277

Means of IFNγ levels were compared using two-way ANOVA with Tukey or Šídák correction for multiple comparisons. Unvac, unvaccinated. DPI, days post infection. HK, heat-killed. 2-Ag, vaccination with CAF01-Cda1+Cda2. 4-Ag, vaccination with CAF01-Cda1+Cda2+Blp4+Cpd1Δ. -, not applicable. *P* < 0.05 was considered significantly different and highlighted with red color.
